# A study on the correlation between MTHFR and folic acid combined with trace elements for the prevention of fetal malformations in the first trimester of pregnancy

**DOI:** 10.1097/MD.0000000000035330

**Published:** 2023-11-03

**Authors:** Hong Zhang, Juan Pan, Haoyu Jiang, Xiaobo Xiong, Lu Huang, Xin Liu, Wei Xin Wangzi, Lida Chen

**Affiliations:** a Department of Clinical Laboratory, Wuhan City Maternal and Child Health Care Hospital, Wuhan, Hubei Province, China.; b Department of Clinical Laboratory, Wuhan Jiangan District Maternal and Child Health Care Hospital, Wuhan Jiangan District Maternal and Child Health Care Planning Service Center, Wuhan, Hubei Province, China

**Keywords:** correlation, fetal malformation, folic acid, MTHFR, trace elements

## Abstract

This study aims to elucidate and examine the intricate interrelation between 5,10-methylenetetrahydrofolate reductase (MTHFR), combined folic acid (FA), and trace element supplementation as a preventive strategy against fetal malformations during the inaugural trimester of pregnancy. Eighty pregnant women selected from our hospital’s early obstetrics department from May 2021 to August 2021. Pregnant women are divided into the MTHFR combined group, FA, and trace element group. Comparing the basic data of patients, analyzing adverse reactions in pregnant women, and total birth risk situation, detecting MTHFR gene polymorphisms, and analyzing the correlation between MTHFR and FA in the prevention of fetal malformations in early pregnancy. Compared with the north, the southern region is more prone to FA deficiency. MTHFR degree of the MTHFR combined group was positively correlated with fetal malformations. The deformity rate was negatively correlated with FA and trace elements. Pregnant women in the first trimester may have fetal malformations, and the malformation rate is negatively correlated with FA and positively correlated with MTHFR level. Importantly, the inverse relationship between FA supplementation and malformation incidence underscores its significance as a preventive measure.

## 1. Introduction

Fetal malformation is a multifactorial and related disorder of the chromosomal or anatomical structure of the fetus within the maternal uterine cavity.^[1–5]^ Looking at global population data, there are approximately 300,000 cases of deformed children for every 10 million people during pregnancy.^[6–8]^ From a comprehensive analysis of regions, medical care, and economics, fetal deformities mainly occur in incompletely developed countries like China, accounting for more than three-quarters of cases.^[9,10]^ With the increasing ecological The trend of diversification and diversification of eating and drinking structure is gradually emerging. The number of deformed fetuses is also increasing year by year.^[11–13]^ Fetal deformities cause death and disability, which has become an important node affecting social stability. Therefore, it is particularly important to find a suitable low-risk method to prevent fetal malformations.

Since the 20th century, experimental and clinical studies have confirmed that fetal malformations are closely related to 5,10-methylenetetrahydrofolate reductase (MTHFR), folic acid (FA), and trace elements, and will affect the fetal condition of early pregnant women.^[14–18]^ Genetic susceptibility genes such as the MTHFR gene may be involved in the occurrence and development of fetal malformations.^[17]^ Based on this foundation, this article takes women in early pregnancy as the starting point to study the value and correlation of MTHFR, FA, and trace elements in preventing fetal malformations, in order to provide reference for fetal assessment during pregnancy. The report is as follows.

## 2. Data and methods

### 2.1. Clinical data

The study was approved by the Ethics Committee of Wuhan Maternal and Child Health Hospital. The study enrolled a total of 80 participants from our institution between May 2021 and August 2021. These participants were evenly divided into 2 groups: the MTHFR combined group and the normal pregnant women group. Informed consent forms were duly signed by the legal guardians of preterm infants. Notably, there were no significant disparities in terms of age and gender among the selected patients (*P* > .05). Subsequently, we conducted a follow-up of discharged patients for a period of time. Further details are available in Table 1

**Table 1:** Compares the general data of the patients.

Group class	Number of cases	Mean (x ± s, age)
The MTHFR combination group	40	29.38 ± 4.29
Normal pregnant women group	40	30.02 ± 3.34
*t*/*χ*^2^	–	–
*P*	–	–

### 2.2. Inclusion criteria

(1) Pregnant women are about 0 to 12 weeks away from delivery; (2) pregnant women aged 22 to 38 years old.

### 2.3. Exclusion criteria

(1) Cardio renal insufficiency and cancer patients should be excluded; (2) patients under the age of 18; (3) patients with genetic or mental illness; (4) patients without informed consent.

### 2.4. Detection methods

The human MTHFR gene was qualitatively detected. Four milliliter of fasting venous blood from all pregnant women and anticoagulation with EDTA for MTHFR gene testing. The polymorphism of MTHFR gene 677 T locus in the DNA of fresh whole blood samples was detected by fluorescent PCR. MTHFR 677 C/T gene detection kit was purchased from Shenzhen Osa Pharmaceutical Co., Ltd., Beijing, China, and the detection instrument was Light Cycler 480 fluorescence quantitative PCR instrument (Roche, Switzerland). Correlation analysis was performed using Spearman correlation analysis.

### 2.5. Statistical methods

Substitute the selected data into SPSS17.0 software for data analysis, the measurement data is represented by (*x* ± s), and the *t* test is performed; the count data is represented by %, and the chi-square test is used. *P* < .05, there is statistical significance.

## 3. Results

### 3.1. Adverse reactions in pregnant women

The adverse reactions of pregnant women in MTHFR combination group and normal pregnant women group are shown in Table 2.

**Table 2:** Adverse reactions: (%).

Group	Low hemoglobin	Body fatigue	Subcutaneous hemorrhage	Total incidence
The MTHFR combination group	3 (7.50)	6 (15.00)	4 (10.00)	13 (32.50)
Normal pregnant women group	1 (2.50)	2 (5.00)	2 (5.00)	5 (12.50)
*χ* ^2^	–	–	–	4.588
*P*	–	–	–	0.032

### 3.2. Fetal birth risk situation

The normal pregnant women group was better than the MTHFR combined group (*P* < .05). Compared with the 2 groups, the low and high risk groups were both 0. There were 25 cases of fetal medium risk in the MTHFR combined group, but none were found in the normal pregnant women group.

### 3.3. Wuhan MTHFR gene polymorphism detection

The MTHFR genotype frequency and allele frequency distribution of Han women in Wuhan area were shown in Table 3. The frequency distribution of MTHFR gene in women in Wuhan has its own characteristics.

**Table 3:** Determination results of FAM and VTC gene polymorphism test in Wuhan region.

Reaction liquid	Gene polymorphisms	FAM channel	VTC channel
MTHFR677	MTHFR677C/C is both pure and wild	Ct value = A = 36	Ct value > 36 or no Ct value
	The MTHFR677C/T heterozygous mutations	Ct value = A = 36	Ct value = A = 36
	Homozygous mutations of MTHFR677T/T	Ct value > 36 or no Ct value	Ct value = A = 36

### 3.4. Lack of FA levels in the north and the south

Compared with the north, the southern region is more prone to FA deficiency (Table 4).

**Table 4:** Folic acid levels in the north and the south.

Group	Low level (<7 mmol/L)	Medium level (<5 mmol/l)	High level (<3 mmol/l)
South	36.18 ± 3.32	116.15 ± 4.24	124.18 ± 5.32
North	31.23 ± 2.17	105.37 ± 4.53	118.43 ± 5.25

### 3.5. Age distribution of people at high risk of FA

**Figure 1.**
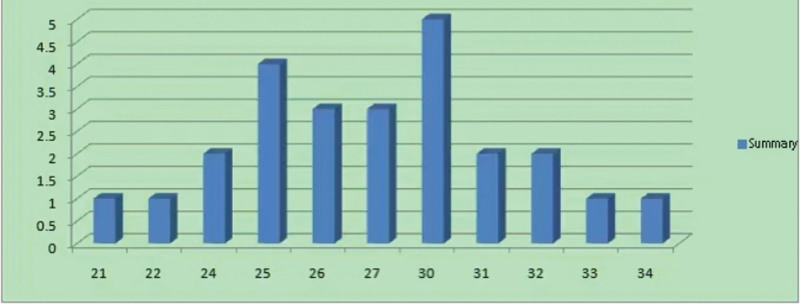
Age distribution of people at high risk of folic acid.

People at high risk for FA are predominantly in the age range between 25 and 30 years (Fig. 1).

### 3.6. Correlation analysis between MTHFR and fetal malformations

Analysis (correlation) of MTHFR and fetal malformations with statistically significant differences between the MTHFR combination groups showed that the MTHFR degree of the MTHFR combined group was positively correlated with fetal malformations (*R* = 0.387, *P* = .253).

### 3.7. Correlation analysis between FA and fetal malformations

The analysis found that the amount of FA and fetal malformations were negatively distributed in the 2 groups (r = −0.517, *P* < .01).

### 3.8. Correlation analysis between trace elements and fetal malformations

Linear analysis showed that the level of trace elements was negatively correlated with fetal malformations (r = −0.383, *P* < .01).

## 4. Discussion

Fetal malformations arise from intricate causes,^[19,20]^ encompassing maternal, fetal (hereditary), and environmental factors. The structure and chromosomal abnormalities of the fetus in the womb are defined as fetal malformations, and the mortality rate of fetal malformations is extremely high.^[21–25]^ It is particularly necessary to intervene in the early stages of pregnancy to improve the quality of the country’s newborn population.

Folic acid (FA), or vitamin B9, is found in many plants, including spinach, corn, and more.^[26–29]^ Folic acid plays a pivotal role in diverse methylation reactions involving amino acids and proteins. As an essential nutrient, its significance for the well-being of pregnant women is self-evident.^[30–33]^ In this study, the selected subjects exhibit varying degrees of FA supplementation, contributing to a reduction in the risk of fetal malformations among pregnant women to a certain extent.

The primary limitation of this study centers on the relatively small sample size and the concentration of main clinical data within our hospital during the period of May to August 2021. Due to the large variety of trace elements, there is no specific correlation between trace elements and fetal malformation in early pregnancy. The future research endeavors will encompass an expanded sample collection from a broader range of sources and will encompass a multi-center study design.

### 4.1. The incidence of fetal malformations in early pregnancy

Pregnant women are in a state of mother–fetal community with multiple needs of nutrients, so the supply to pregnant women should be more than that of ordinary women. The average incidence of fetal malformations in pregnant women (12 weeks) found in domestic and foreign studies was 13.6%. The pregnant women in this study were influenced by a convergence of multiple factors impacting fetal morphological development.

### 4.2. The effect of MTHFR on pregnant women

This analysis unveils a connection between FA and hemoglobin levels in early pregnancy, which could potentially lead to fetal malformation or miscarriage. MTHFR is associated with fetal malformations. Studies have shown that MTHFR gene mutation is an independent risk factor for fetal malformation. Correlation assessments of MTHFR gene polymorphism in the Wuhan region highlight distinct frequency distributions within local women. Thus, targeted regulation could mitigate the risk of congenital anomalies.

### 4.3. Effects of FA on pregnant women

In addition, the detection rate of fetal malformation in both groups was negatively correlated with the level of FA. Therefore, it is recommended that early pregnant women, especially those with anemia, supplement FA early to increase the fetal eugenic rate.

### 4.4. The effect of trace elements on pregnant women

Trace elements such as zinc, selenium, calcium, iron, copper, and magnesium are essential for the body, and even more so in the body of pregnancy. Studies underscore the pivotal role of trace elements (such as zinc, selenium, iron) in the onset of fetal malformations during early pregnancy. Zinc, implicated in the processing and synthesis of over 200 enzymes and proteins, stands as a pivotal component of cell mitosis, profoundly influencing the formation and progression of bodily organs. Inadequate zinc levels can severely compromise proper organ development, potentially leading to deformities. Similar significance is attributed to other trace elements, all of which wield substantial influence in the course of human growth.

## 5. Conclusion

In the early stages of pregnancy, pregnant women may experience fetal malformations, with the malformation rate being inversely correlated with folate levels and positively correlated with MTHFR levels. Trace elements serve as protective factors; specifically, the more ample the trace element presence, the lower the likelihood of fetal malformations. Of course, the body is a multi-complex, and the study of nutrients alone fails to fully explain the rationale. Subsequent healthcare professionals should conduct comprehensive evaluations of maternal conditions.

## Author contributions

**Conceptualization:** Hong Zhang, Juan Pan.

**Data curation:** Juan Pan.

**Formal analysis:** Haoyu Jiang, Xin Liu.

**Investigation:** Xiaobo Xiong, Lida Chen.

**Methodology:** Lu Huang, Xin Liu, Lida Chen.

**Software:** Wei Xin Wangzi.

**Supervision:** Wei Xin Wangzi, Lida Chen.

**Visualization:** Wei Xin Wangzi, Lida Chen.

**Writing – original draft:** Hong Zhang.

**Writing – review & editing:** Hong Zhang, Juan Pan.
